# Progress in Surgery

**Published:** 1897-10-16

**Authors:** 


### PROGRESS IN SURGERY.

### GENITO-URINARY SURGERY.

**The Renal Circulation.**—M. Destot has recently made observations on the circulation of the kidneys by means of metallic injections and radiography. The veins communicate freely even from lobule to lobule, but the arteries do not anastomose. The ventral and dorsal segments of the kidney are supplied by two separate arterial branches, hence incisions should be made midway in the convex border.[1]

**Renal Disease in Relation to Surgical Operation.**—The importance of adequate kidneys is emphasised by Dr. Tuttle (Mass.).[2] The importance of the estimation of the amount of urea in the urine is pointed out by him, as well as the significance of urine of a constantly low specific gravity. A reduction of 50 per cent. in the amount of urea excreted should contra-indicate severe operation, which would itself throw much increased work upon the kidneys.

The following points are borrowed from a lecture by Professor Obalinski.[3] We only nowadays sacrifice a kidney by nephrectomy when it contains within itself a focus which may be injurious to the organism, *i.e.*, when a malignant growth or primary tuberculosis are positively diagnosed. When an enlarged, hard kidney can be felt before operation, it is probably functionless or nearly so, and the other kidney is capable of performing the functions of both. Catheterisation of the ureters is of little practical value in determining nephrectomy, since a diseased kidney may still be capable of doing the work of two. Where, in the kidney to be operated on, we suspect that there is still a certain functional capacity we should never sacrifice it, but perform nephrotomy.

The adequacy of the remaining kidney after nephrectomy cannot be always foreseen. Indeed, a kidney which is to all macroscopic and microscopic appearances healthy, may be for some obscure reason functionless, and fatal anuria occur. In 1 in 300 persons one kidney alone exists. Practically exploratory incision is the only means of recognising renal abnormalities; cystoscopy and ureteral catheterisation are unreliable.[4]

**Rupture of Kidney.**—A lecture on this subject has been communicated by Mr. Herbert Page,[5] based upon a case which required nephrectomy after drainage by gauze had been used in vain. There are two great dangers of ruptured kidney—(*a*) hæmorrhage, (*b*) sepsis. Primary nephrectomy for ruptured kidney prevents sepsis; secondary nephrectomy when sepsis has developed has a mortality twice as great as primary nephrectomy.

**Moveable Kidney.**—Of some 38 bodies examined by Dr. Francis Watson[6] 20 per cent. had moveable kidney. In the opinion of Dr. John Bryson (St. Louis) the real pathological condition is a disturbance in the condition of the fatty capsule, with or without general enteroptosis. The pain is due to traction and twisting of the structures at the root of the kidney with congestion of the kidney itself. Rotation of the pedicle is particularly painful.

A review of the operations for moveable kidney is made by Dr. Stinson (San Francisco), who also gives a method of his own, which, however, departs but little from the practice of some other surgeons.[7] The methods are—(*a*) Simple suture, including kidney substance and adjacent fasciæ and muscles, using four to six sutures; (*b*) partial stripping off of capsule proper with stitching of the rawed surface of the kidney to the raw edges of the lumbar muscles; (*c*) suture of fibrous capsule below, and the upper half of the capsule is slit up at the posterior edge of the organ, and the whole wound packed with gauze and allowed to granulate up from the bottom; (*d*) fixation of upper part to the periosteum of twelfth rib, packing the rest with iodoform gauze, and allowing the whole wound to granulate. Dr. Stinson holds, contrary to the opinion of many surgeons of repute, that the sutures should not traverse the secreting structure of the kidney; he agrees with many in drawing in the slack of the fatty capsule.

**Hydronephrosis.**—The tendency is to avoid nephrectomy for hydronephrosis whenever it is possible to leave the organ, no matter how little secreting structure exists in it. The enormous sac speedily contracts after the relief of urinary pressure, and the function of the remaining secreting structure is restored. Bardenheuer established an anastomosis between the ureter and the most dependent portion of the sac in one case, and in another cured the kink in the renal pelvis by plastic operation similar to that of Heineke-Mikulicz and Fenger for pyloric stenosis. According to Mr. Bland Sutton, many large examples of hydronephrosis probably begin during intra-uterine life. He gives a figure illustrating such a congenital defect.[8]

**Calculus.**—Attention is called by Dr. John Duncan, Edinburgh, to occasional occurrence of sciatica, due to renal calculus. He also describes a case in which over a thousand minute calculi were found.[9]

**Tuberculosis.**—A paper was read by Dr. Willy Mayer urging early nephrectomy for tuberculosis of the kidney.[10] This was illustrated by a case which showed some bladder changes by cystoscopy, and some stricture of the ureter by catheterisation. The urine passed contained tubercle bacilli, but by catheterisation it was found that the urine from the left ureter was healthy. The patient recovered after nephrectomy, and the bladder improved. The value of cystoscopy is insisted upon.

Mr. Maylard, Glasgow, reported two successful cases of nephrectomy for tuberculous disease, and expressed a strong opinion that nephrotomy is useless for tuberculous kidneys, because the foci of disease are usually multiple.

**Cysts of the Kidney.**—This subject has been well reviewed by Dr. David Newman, with descriptions of typical examples.[11] He makes the following groups:

(*a*) Cystic disease in adults, associated with chronic nephritis; (*b*) congenital cystic kidney; (*c*) paranephric cysts; (*d*) parasitic cysts—true hydatids.

Specimens of chronic nephritis frequently show cysts of various sizes, but generally speaking small, and usually most abundant in the limiting zone between cortex and medulla. Plugs of colloid material may be seen blocking the uriniferous tubules. Dr. Newman regards the condition as true retention cysts due to localised interstitial nephritis. Other views are that the cysts occur in embryonic rudiments of the Wolffian body by congenital defect, and in support of this it is noted that there are sometimes associated congenital defects such as arrested development of the heart; or they may be due to congenital atresia of the canaliculi (Virchow).

The condition is frequently double, and occurs in adults with concomitant signs like those of Bright's disease, such as casts in the urine, hæmaturia, cardiac hypertrophy, and increased arterial tension. The patient's aspect is like that of one with Bright's disease, and he may die with coma or convulsions. In the diagnosis of the disease it must be remembered that there is gradual enlargement of the kidneys, often both of them, without change in general form, but perhaps with perceptible nodulation on the surface. The tumours do not get smaller as in hydronephrosis. The urine may be normal, but commonly resembles that of Bright's disease; at the same time, dropsy does not occur, and hæmaturia may be a prominent symptom.

The appearance of congenital cystic kidneys is well known. The cysts may be retention cysts from embryonic nephritis or blockage of the tubules by uric acid, or may be due to maldevelopment. Surgical treatment is rarely indicated.

Paranephric cysts are formed outside the capsule of the kidney, and do not contain urinary constituents.

Parasitic cysts are represented by hydatids, which are rare in the British Isles. They are apt to resemble ovarian cysts, but usually let the cat out of the bag by discharging their daughter cysts per urethram. Only some 6 to 8 per cent. of hydatid cysts are renal, and 80 per cent. of these discharge their contents into the renal pelvis, sometimes with marked renal colic. The urine should be very carefully examined for hooklets in these cases.

**Suppurations in the Urinary Apparatus.**—Observations upon this subject have been made by Hugo Maass[12] (Berlin) and by Mr. Reginald Harrison.[13] The fatty capsule of the kidney is predisposed to suppuration by reason of its loose cellular tissue. The abscesses are usually behind the kidney when the fatty capsule is most developed. Pyelonephritis and pyelitis are frequent causes, but in many cases the ætiology is obscure. It may follow inflammations of the pelvic fascia after child-birth or operations on the bladder. It is very doubtful whether perirenal abscess is ever primary.

Mr. Harrison points out that many cases of so-called gleet represent a far more extensive invasion than is generally supposed to be the case. Many gleets are merely re-infections of the urethra from a bladder which serves the purpose of a medium for bacterial cultivation. The invasion of the bladder in this way is not uncommon, and is not necessarily accompanied by acute symptoms. Gonoccocci may be found in vesical urine in cases of gonorrhœa. For this condition antiseptic irrigation of the bladder should be employed, such as weak Condy's fluid. If, when voiding urine, the patient suddenly interrupts the flow by penile compression the lacunæ will be thus opened out and flushed. For aborting infectious inflammations perchloride of mercury (1 in 10,000) is most useful. After its use the bladder may be filled with a strained solution of albumin, which counteracts the perchloride and prevents pain. The internal administration of bactericides may be advantageously combined with the local treatment, such as sandal-wood, copaiba, cubebs, &c. Boric acid, salol, quinine, salicylate and benzoate of soda are valuable. Urinary fistulæ may be sometimes caused to heal by flushing, first of all injecting the bladder and then allowing the patient to pass urine naturally, when some of the solution comes through the fistula.

[1] Lyon Médical, 1896, Nos. 49 and 50. [2] Medical Times and Register, April 17, 1897. [3] Gazeta Lekarska, Nos. 40 and 41, 1896. [4] Therapeutical Gazette, April 15, 1897. [5] Clinical Journal, Nov. 25, 1896. [6] N. Y. Med. Record, May 15, 1897. [7] N. Y. Med. Record, April 17, 1897. [8] Clinical Journal, July 14, 1897. [9] Scottish Med. and Surg. Journal. [10] N. Y. Med. Record, Jan. 9, 1897. [11] Glasgow Med. Journal, July, 1897. [12] Sammlung Klinische Vorträge, No. 170, 1896. [13] Lancet, June 26, 1897.

### RECTUM AND ANUS.

**Atony of Rectum and Anus.**—This is the title of a paper by Dr. William Bodenhamer, New Rochelle, N.Y.[1] He points out the difference between atony and constipation—the one a disease, the other an effect having many causes. Prolonged constipation is, however, itself a cause of atony by over-distension of the rectum; but rectal distension may itself be caused by atony, and so the vicious circle works. Such primary atony may result from pressure of the fœtal head during labour, from ovarian tumours, displacements of the uterus, and the like; in these the disease is a local one—true atony of the rectum. The most common cause of atony of the rectum is habitual disregard to the calls of Nature. It is also frequently caused by the enema habit when hot water is used. Cold water does not seem to have so great a tendency to cause atony. Flushing with large quantities of water is distinctly harmful. The chief symptom of atony is a feeling of fulness and distension which is not properly relieved by defæcation, confirmed by feeling a fæcal mass in the rectum. Sometimes the mucous membrane is smooth, and at other times one finds it thrown into pendant folds.

In the treatment regular habits of evacuation should be enforced, aided for a time, if necessary, by small injections of cold water. In more obstinate cases nux vomica, and injections of powerful astringents and tonics become necessary. Gentle aperients may have to be given as adjuvants. The astringent enemata should be small in quantity, say five or six ounces, and be given after the bowels have acted. They comprise galls, tannic acid, oak bark, rhatany, alum, &c. Hard fæcal masses must, of course, be removed by mechanical means.

Atony of the sphincters occurs occasionally in old age, is often associated with prolapsus ani, and has many causes, both local and constitutional, the removal of which cures the disease.

**Stricture of the Rectum.**—A new method of operation—"external rectotomy"—was described by E. Sonnenburg (Berlin) in the "Medical Annual" for 1897. The methods hitherto in use have been—(1) Dilatation by bougies; (2) internal rectotomy; (3) colotomy; (4) extirpation; (5) Pean's method—*i.e.*, by division of skin and posterior wall of the rectum longitudinally, and stitching up transversely. In Sonnenburg's operation the stricture is divided from without inwards after removal of part of the sacrum, as in Kraske's operation. Recovery takes a long time, but the sphincter is preserved, and the results are good. Bougies are subsequently necessary. No suturing of the rectum is employed, the wound being allowed to granulate.[2]

Another new method of treating stricture of the rectum is that described by Dr. J. B. Bacon, Chicago.[3] It consists in first passing a seton of strong silk through the rectal wall just above the sphincter, then up through the perirectal tissue outside the stricture, between it and the coccyx. The thread is then brought through the strictured gut, and tied to the other end. This seton must be left in for three months to establish a continuous mucous tract. The patient is again anæsthetised, and the stricture divided through into the mucous tract by means of a Paqulin's cautery on a director.

A series of cases of excision of the rectum for syphilitic stricture, villous tumour, and cancer has been published by Mr. F. T. Paul, of Liverpool.[5] The cases include a large villous tumour, a villous tumour simple in some parts, malignant in others. He holds that colloid cancer is decidedly more malignant than cylindrical-celled. About three inches of the bowel can be removed by perineal incision. Removal of the posterior wall of the vagina gives more room. Small growths may be removed by posterior median proctotomy, with subsequent suture. Kraske's operation is useful for extensive growths. Mr. Paul thinks it a mistake to leave a part of the circumference of the bowel; if the bowel cannot be restored to perfect continuity by suture, he thinks much better results are got with the glass tube. Cancer is apt to occur earlier in the rectum than in any other site, but there are few parts of the body where better results can be obtained by operation. The mortality from the operation is 14 per cent. Mr. Jackson Clarke has published a successful case of Kraske's operation.

The question of the best treatment for cancer of the rectum has been recently discussed before the French Chirurgical Society.[6 7] In many cases preliminary inguinal colotomy is recommended, advantage being taken at the same time of the opportunity of exploring the upper part of the rectum and sigmoid. This practice of making an artificial anus before proctectomy seems to gain much favour with French surgeons.

One great difficulty in the treatment of cancer of the rectum is the fact that the lymphatic glands may be early infected, and are almost out of reach of any form of operation.

[1] N.Y. Med. Journ., June 19, 1897. [2] Medical Annual, 1897. [3] Mathew's Quarterly Journal, Jan., 1897. [4] Lancet, June 26, 1897. [5] Lancet, July 10, 1897. [6] Med. Week, June 25, 1897. [7] Med. Week, July 9, 1897.

